# Membrane-Permeable Octanoyloxybenzyl-Masked cNMPs As Novel Tools for Non-Invasive Cell Assays

**DOI:** 10.3390/molecules23112960

**Published:** 2018-11-13

**Authors:** Alexandra Ruthenbeck, Elisa Marangoni, Björn-Ph. Diercks, Aileen Krüger, Alexander Froese, Nadja I. Bork, Viacheslav O. Nikolaev, Andreas H. Guse, Chris Meier

**Affiliations:** 1 Organic Chemistry, Department of Chemistry, Faculty of Sciences, University of Hamburg, Martin-Luther-King-Platz 6, D-20146 Hamburg, Germany; ruthenbe@chemie.uni-hamburg.de (A.R.); elisa.marangoni@studenti.unicam.it (E.M.); 2 School of Pharmacy, Medicinal Chemistry Unit, University of Camerino, via S. Agostino 1, 62032 Camerino, Italy; 3 University Medical Center Hamburg-Eppendorf, Martinistraße 52, 20246 Hamburg, Germany; b.diercks@uke.de (B.-P.D.); Krueger-Aileen@web.de (A.K.); a.froese@uke.de (A.F.); n.bork@uke.de (N.I.B.); v.nikolaev@uke.de (V.O.N.); guse@uke.de (A.H.G.)

**Keywords:** cyclic nucleotide monophosphate, bio-reversible protection, acyloxybenzyl phosphate ester

## Abstract

Adenine nucleotide (AN) 2nd messengers, such as 3′,5′-cyclic adenosine monophosphate (cAMP), are central elements of intracellular signaling, but many details of their underlying processes remain elusive. Like all nucleotides, cyclic nucleotide monophosphates (cNMPs) are net-negatively charged at physiologic pH which limits their applicability in cell-based settings. Thus, many cellular assays rely on sophisticated techniques like microinjection or electroporation. This setup is not feasible for medium- to high-throughput formats, and the mechanic stress that cells are exposed to raises the probability of interfering artefacts or false-positives. Here, we present a short and flexible chemical route yielding membrane-permeable, bio-reversibly masked cNMPs for which we employed the octanoyloxybenzyl (OB) group. We further show hydrolysis studies on chemical stability and enzymatic activation, and present results of real-time assays, where we used cAMP and Ca^2+^ live cell imaging to demonstrate high permeability and prompt intracellular conversion of some selected masked cNMPs. Based on these results, our novel OB-masked cNMPs constitute valuable precursor-tools for non-invasive studies on intracellular signaling.

## 1. Introduction

Among cNMPs, the ubiquitous second messengers 3′,5′-cyclic adenosine monophosphate (cAMP) and 3′,5′-cyclic guanosine monophosphate (cGMP) constitute the most prominent examples, but further cNMPs, such as 3′,5′-cyclic uridine monophosphate (cUMP), have been reported as related to signaling processes [1]. Signaling by cNMPs proceeds via two general pathways. The first relies on direct binding and regulation of distinctive cyclic nucleotide-gated (CNG) ion channels. The second mechanism is based on the activation of protein kinases A (PKA) or G to further transduce the signal [1,2,3,4]. Activated PKA/G promotes phosphorylation of a variety of proteins, which can be involved in the regulation of metabolic processes, muscle contraction, and gene transcription [4]. In contrast, signaling through CNG channels allows a faster processing and implementation of increased cNMP levels. The ion channels are generally non-selective for cations. However, the entry of sodium ions (Na^+^) depolarizes the membrane which promotes the combined influx of Ca^2+^. Additionally, voltage-gated Ca^2+^ channels open in response to membrane depolarization and thus enhance the Ca^2+^ signal further [2,3]. Signals of cAMP and cGMP are ceased through their degradation by phosphodiesterase to AMP and GMP, respectively [2,3].

Based on the ubiquitous involvement in cellular processes, the role of cNMP signaling in the context of inflammation and regulation of immune response constitutes a research field of rising interest. It was, for example, found that regulatory T cells (T_reg_) exert their suppressive effect on effector T cells (T_eff_) through increasing concentrations of cAMP. The rise of cAMP levels in T_eff_ could either be induced via a paracrine mechanism, or by a direct transfer of cAMP via gap junctions between T_reg_ and T_eff_ [5,6]. However, the transfer of cAMP between a pair of T cells cannot yet be visualized directly, and the underlying mechanisms allowing T_reg_ cells to produce such significantly higher cAMP levels than T_eff_ cells have not been elucidated to date. A loss of the immuno-suppressive mechanism could contribute to the generation of autoimmune reactions. Understanding the role of cNMPs in inflammation and T cell regulation and identifying the associated molecular pathways thus could enable the identification of novel targets in the treatment of autoimmune disease.

However, cell-based studies on 2nd messengers are in our experience difficult to perform as application of the highly polar compounds is carried out via effortful, single-cell preparative methods like electroporation, microinjection, or patch clamp. These methods require highly trained staff, careful preparation and significant amounts of time in advance of each experiment. At the same time, the invasive application raises the potential for interfering artefacts or false-positives [7]. Thus, membrane permeable, bio-reversibly modified chemical derivatives of second messengers are highly desirable compounds to circumvent these drawbacks.

**Figure 1:**
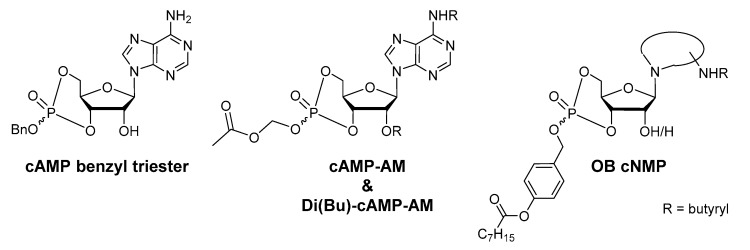
Masked cAMP derivatives. Left: Benzyl triesters of cAMP reported by Engels et al. [8,9]. cAMP-AM derivatives introduced by Schultz et al. (middle [12,13], Right: structure of the OB-masked cNMPs disclosed here.

First reports on phosphate-protected cNMPs by Gillen and Nagyvary on alkyl phosphotriesters of cAMP were followed by Engels et al. who prepared cAMP and cGMP derivatives carrying different benzyl groups including photo-cleavable moieties at the phosphate (Figure 1) [8,9,10,11]. Hughes et al. reported the synthesis and biologic evaluation of *N*^6^,*O*^2^-dibutyryl cAMP-acetoxymethyl (AM) ester (Figure 1) [12]. The synthesis started from *N*^6^,*O*^2^′-dibutyryl cAMP, which was converted with AM bromide (AM-Br) under DIEA-basic conditions in CH_3_CN over 4 days at room temperature (rt) to give the desired product in low yield after demanding chromatography [12]. The obtained masked nucleotide was used in whole-cell incubation studies where an activation of PKA proceeded, but only 15 min after the addition of di(Bu)cAMP-AM (10 µM) to the cell medium [12]. In a further study, Schultz et al. synthesized cAMP-AM [13]. Despite a PKA activating effect of cAMP-AM, the researchers found that cAMP-AM was less potent than di(Bu)cAMP-AM and was metabolized so rapidly that it gave only transient signals [13]. The synthesis of AM-esters of cCMP and cUMP has also been reported [14]. However, cNMP-AMs are in our experience not applicable for all cell types, seemingly due to their insufficient chemical stability. Further, the variability of the described synthesis approaches, which started from the respective cNMP, is limited by the availability of these.

The acyloxybenzyl (AB) group constitutes another successfully applied bio-reversible masking group apart from AM esters. It was introduced originally for nucleoside monophosphate (NMP) prodrugs and transferred on nucleoside diphosphates (NDPs) and nucleoside triphosphates (NTPs) [15,16,17,18,19]. The respective prodrugs were shown to efficiently diffuse across cell membranes and release the corresponding nucleotide intracellularly upon enzymatic activation [16]. Further, the concept was expanded on compound classes like sugar nucleotides [20]. The broad synthetic applicability of the AB masking group is complimented by meeting the essential features of a prodrug concept: (i) high chemical stability; (ii) efficient enzymatic activation; and (iii) sufficient lipophilicity to enable the passage through cell membranes. Moreover, the two-part composition of the AB-masks allows variation of e.g., lipophilicity or enzymatic cleavability which adds further versatility to the concept [15,16,17,21,22,23]. Consequently, the AB-concept was investigated for expansion on cNMPs to provide tools for studies on cellular effects, e.g., calcium signaling in bulk settings on a variety of cells and without the need for example of microinjection. As a first example, an octanoyloxybenzyl (OB)-mask was used here since previous studies showed a good chemical stability and sufficiently high lipophilicity of respectively masked di- and triphosphates (Figure 1) [13,14,15].

## 2. Results and Discussion

Inspiration for the synthesis approach towards OB-masked cNMPs was drawn from own studies on the synthesis of non-symmetric phosphoramidites (PAs) that included an unprotected nucleoside moiety. In these cases, side reactions were repeatedly observed amongst which the formation of phosphites was prominent. These phosphites were concluded to result either from an intermolecular or an intramolecular substitution of the remaining *N*-di*iso*propyl group by a second nucleosidic hydroxy-group. In the latter case, a cyclic nucleoside phosphite would constitute the reaction product, and further oxidation would result in a cyclic nucleoside monophosphate derivative.

Based on this hypothesis, we set up a synthesis involving different nucleosides and an OB-masked phosphordiamidite (OBPA_2_).

### 2.1. Preparation of AB-Masked cNMPs

#### 2.1.1. Synthesis of Starting Materials

**Scheme 1:**
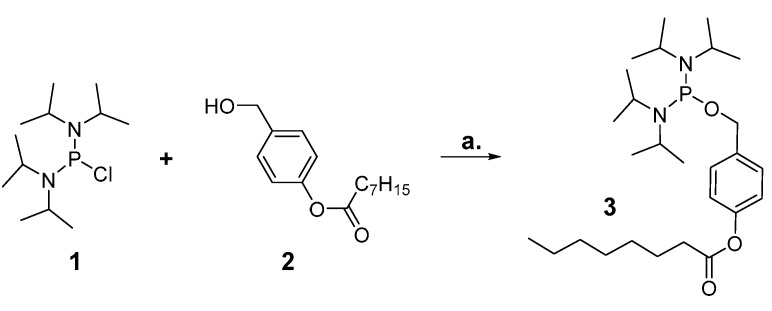
Synthesis of OBPA_2_ **3**: **a.** 1 equiv. **1**, 1 equiv. **2**, 1.3 equiv. TEA, THF, rt, 18 h, **3**: 71%.

The synthesis of bis(*N*,*N*-di*iso*propylamino)-4-octanoyloxybenzyl phosphordiamidite (OBPA_2_) was adapted from Weinschenk et al. who used the building block for the synthesis of non-symmetric phosphoramidites [24]. The reaction was performed as described in the literature starting from bis(*N*,*N*-di*iso*propylamino)chlorophosphine **1** and 4-(hydroxymethyl)phenyloctanoate **2**, and yielded OBPA_2_ **3** in 71% (Scheme 1).

**Scheme 2:**
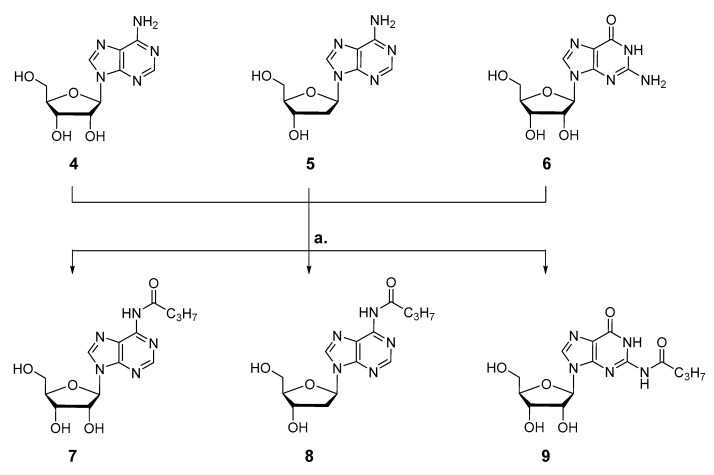
Synthesis of *N*-butyryl nucleosides **7**–**9**: **a.** first 2.1–9.0 equiv. TMSCl, 0 °C to rt, 18 h, second 1.1 equiv. butanoyl chloride, pyridine/THF or pyridine/CH_2_Cl_2_, rt, 5–18 h, third 1 M aq. HCl or CH_3_OH, rt, 5 min or 18 h, **7**: 61%, **8**: 32%, **9**: 48%.

The nucleosides adenosine (A, **4**), 2′-deoxyadenosine (dA, **5**), and guanosine (G, **6**) were mono-*N*-butyrylated to add further lipophilicity to the envisaged OB-cNMPs. *N*^6^-Butanoyl-adenosine **7** was synthesized starting from adenosine **4** via transient silylation of all hydroxy-groups to selectively introduce the butyryl moiety at the *N*^6^-position [25]. The desired protected nucleoside **7** was obtained in 61% yield after automated RP flash column chromatography following the procedure described in the literature [25]. The yield was limited by the formation of *N*,*N*-diacylated adenosine and partial cleavage of the glycosidic bond during concentration of the crude reaction mixture. Analogously, *N*^6^-butanoyl-dA **8** and *N*^2^-butanoyl-G **9** were synthesized starting from the respective nucleosides and obtained in yields of 32% and 48%, respectively (Scheme 2).

#### 2.1.2. Syntheses of OB-cNMPs

First, a model system was set up to study course and outcome of the phosphitylation in more detail. For this served the reaction towards octanoyloxybenzyl-masked cUMP (OB-cUMP, **10**) since syntheses involving uridine commonly go along with little or no side reactions and complications.

**Scheme 3:**
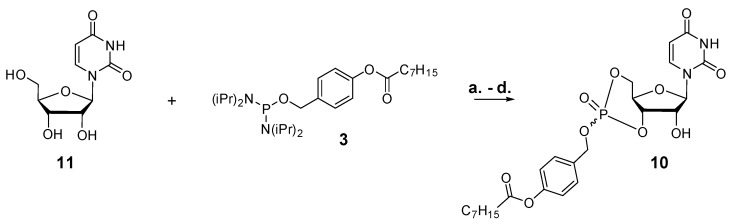
**a.** 1.1 equiv. **3**, 2.2 equiv. DCI (0.25 M in CH_3_CN), 1.5 equiv. *t*BuOOH (5.5 M in *n*-decane), CH_3_CN/DMF 5:4, 0 °C to rt, 60 min, **10**: 13%. **b.** 1.1 equiv. **3**, first portion of 1.3 equiv. DCI (0.25 M in CH_3_CN), second portion of 1.3 equiv. DCI (0.25 M in CH_3_CN), 1.5 equiv. *t*BuOOH (5.5 M in *n*-decane), CH_3_CN/DMF 5:1, rt, 60 min, **10**: 15%. **c.** 1.1 equiv. **3**, first portion of 1.3 equiv. DCI (0.25 M in CH_3_CN), second portion of 1.3 equiv. BTT (0.3 M in CH_3_CN), 1.5 equiv. *t*BuOOH (5.5 M in *n*-decane), CH_3_CN/DMF 5:1, rt, 60 min, **10**: 13–19%. **d.** 1–1.2 equiv. **3**, first portion of 1–1.2 equiv. saccharin and 1–1.2 equiv. 1-methylimidazole, second portion of 1–1.2 equiv. saccharin and 1–1.2 equiv. 1-methylimidazole, 1.5 equiv. *t*BuOOH (5.5 M in *n*-decane), CH_3_CN/DMF 5:1, rt, 60 min to 72 h, **10**: 16–19%.

The starting conditions for the synthesis of OB-cUMP **10** were adapted from the approaches towards non-symmetric phosphoramidites [24]. 4,5-Dicyanoimidazole (DCI, 0.25 M in acetonitrile (CH_3_CN)) was used as activator for the PA-coupling, and *t*BuOOH (5.5 M in *n*-decane) served as oxidizing agent. The reaction was carried out in a mixture of CH_3_CN and DMF due to the limited solubility of uridine **11** in CH_3_CN (Scheme 3).

In the first attempt, a solution of nucleoside **11** and OBPA_2_ **3** was treated with 2.2 equivalents of DCI that were added dropwise at 0 °C. The reaction mixture was allowed to warm to rt and stirred 30 min more before *t*BuOOH was added for oxidation. After further 30 min, all volatile components were removed under high vacuum and the crude reaction mixture was purified by automated RP flash column chromatography. The masked cyclic nucleotide **10** was obtained as a mixture of two diastereomers in a yield of only 13% (Scheme 3).

**Figure 2:**
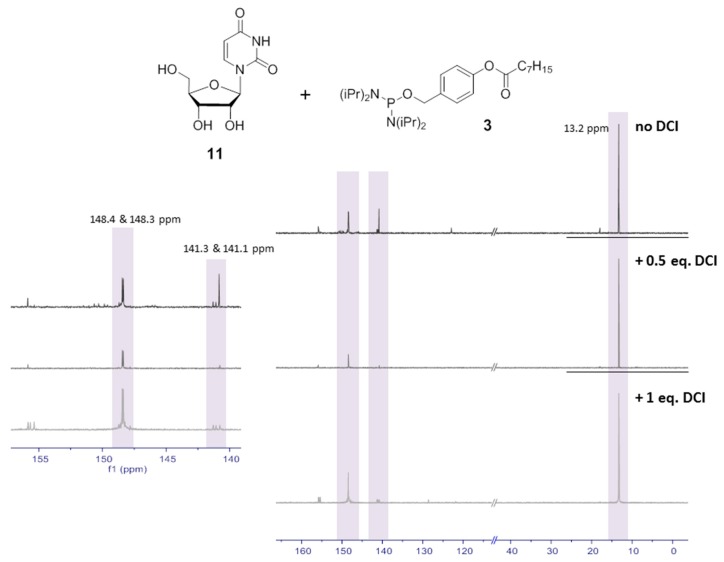
^31^P-NMR spectra (CH_3_CN-d_3_, 162 MHz, 25 °C, shifts δ in ppm) of the reaction monitoring between uridine **11** and OBPA_2_ **3**.

Prompted by the surprisingly low yield, the reaction course was studied ^31^P-NMR spectroscopically (Figure 2). Interestingly, upon just mixing uridine **11** and OBPA_2_ **3**, the diamidite was converted almost quantitatively and a signal at 13.2 ppm formed (Figure 2). Comparison with literature-data indicated that this signal likely corresponded to an activated amidite [26]. Furthermore, signals in the range of phosphoramidites (148.4 & 148.3 ppm) and phosphites (141.3 & 141.1 ppm) were formed already as well (Figure 2, top). The addition of DCI then promoted the formation of the intermediate phosphoramidite (middle and bottom), but formation of the anticipated cyclic phosphite seemed to occur only at low proportion. Steric hindrance in the attack of the 3′-hydroxy group on the phosphorous atom or an insufficient nucleophilicity may be the reasons that led to an insufficient formation of the cyclic phosphite.

The synthesis of OB-cUMP **10** consequently was repeated with several changes to the protocol in order to evaluate the influence of the reaction conditions and in particular the impact of the activator (Scheme 3).

In a second approach, the ratio between CH_3_CN and DMF was altered to 5:1 and the mode of addition of reagents was changed. This time, the nucleoside was placed in the reaction flask and dissolved in CH_3_CN/DMF 5:1. A separate solution of OBPA_2_ **3** in CH_3_CN and one equivalent of DCI were added slowly and dropwise to nucleoside **11** at rt. Once the addition was completed, a second equivalent of DCI was added, and the reaction mixture stirred at rt for 60 min. After the successive oxidation and final purification, OB-cUMP **10** was obtained in 15% yield. Consequently, the outcome of the reaction was almost identical to that of the attempt before.

**Scheme 4:**
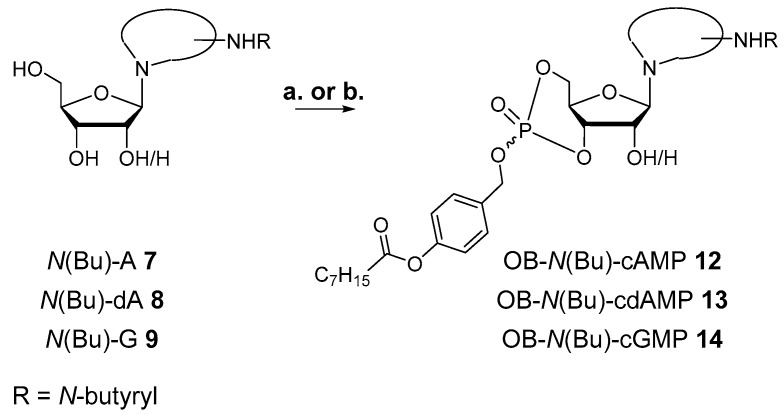
Syntheses of and *N*-butyrylated OB-cNMPs **12**–**14**: To **12**: **b.** 1.1 equiv. **3**, first portion of 1.3 equiv. DCI (0.25 M in CH_3_CN), second portion of 1.3 equiv. BTT (0.3 M in CH_3_CN), 1.5 equiv. *t*BuOOH (5.5 M in *n*-decane), CH_3_CN/DMF 3:1, rt, 60 min, **12**: 14%. To **13**: **a.** 1.1 equiv. **3**, first portion of 1.3 equiv. DCI (0.25 M in CH_3_CN), second portion of 1.3 equiv. DCI (0.25 M in CH_3_CN), 1.5 equiv. *t*BuOOH (5.5 M in *n*-decane), CH_3_CN/DMF 1:1, rt, 60 min, **13**: 13%. To **14**: **a.** 1.1 equiv. **3**, first portion of 1.5 equiv. DCI (0.25 M in CH_3_CN), second portion of 1.5 equiv. DCI (0.25 M in CH_3_CN), 1.5 equiv. *t*BuOOH (5.5 M in *n*-decane), CH_3_CN/DMF 1:1, rt, 60 min, **14**: 4%.

Next, the activator was changed to 5-(benzylthio)-1*H*-tetrazole (BTT) which displays a higher acidity and lower nucleophilicity than DCI, and the reaction protocol was varied as follows: uridine **11** was dissolved in CH_3_CN/DMF 5:1 and successively treated with a solution of OBPA_2_ **3** in CH_3_CN and one equivalent of DCI. Both reagents were added in small portions over a period of 30 min. Upon completed addition, the reaction mixture was stirred another 30 to 120 min at rt. Then, one equivalent of BTT was added slowly and dropwise over 10 min, and the reaction was kept stirring for further 15 to 60 min. After the subsequent oxidation and final purification, OB-cUMP **10** was isolated in yields between 13–19% which again constituted no significant improvement of the reaction outcome. Lastly, an alternative activator-system composed of saccharine and 1-methylimidazole was tested as it was reported to efficiently mediate reactions between PAs and poorly nucleophilic alcohols like tertiary alcohols [27]. For this, saccharine and 1-methylimidazole were dissolved in a 1:1 ratio in CH_3_CN prior to the reaction to generate the activating salt. Simultaneously with OBPA_2_ **3**, one equivalent of the activator salt was added to a solution of uridine **11** in CH_3_CN/DMF 5:1. Upon completion of the first addition, the reaction mixture was stirred for 30 min, then treated with a second equivalent of the activator solution and successively stirred for another 60 min to 72 h. After oxidation and automated RP column chromatography, the desired product **10** was obtained in yields between 16–19%. Monitoring of the reaction course via ^31^P-NMR spectroscopy indicated an incomplete activation of OBPA_2_ **3** even after 72 h. Further, the activated intermediates were again not converted efficiently to the desired phosphite as concluded from the persistence of the respectively attributed phosphorous signals [27]. This alternative approach thus constituted no improvement in comparison to the previous protocols. In summary, the isolation of OB-cUMP **10** succeeded repeatedly despite low yields, and thus the reaction protocols were transferred on the *N*-butyrylated nucleosides **7**–**9** (Scheme 4).

**Scheme 5:**
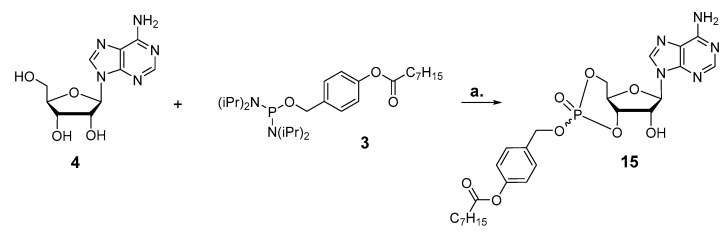
Preparation of OB-cAMP **15**: **a.** 1.1 equiv. **3**, 2.4 equiv. DCI (0.25 M in CH_3_CN) added in two portions, 1.5 equiv. *t*BuOOH (5.5 M in *n*-decane), CH_3_CN/DMF 1:1, rt, 60 min, **15**: 12%.

The *N*-butyrylated OB-cNMPs **12**–**14** were prepared following the synthesis protocols b. and c. described above. The yields obtained for OB-*N*(Bu)-cAMP **12** and OB-*N*(Bu)-c(dAMP) **13** were in a similar range as found for OB-cUMP **10** with 14% and 13%, respectively (Scheme 4). The reaction towards OB-*N*(Bu)-cGMP **14** proceeded to a lower extent and accordingly, the desired product was isolated in a comparably low yield of 4% (Scheme 4). Interestingly, in the case of **14**, the formation of only one of the two possible diastereomers seemed favored as crude ^31^P-NMR spectra indicated. Here, only one of the possible two phosphate signals was prominent, and consequently, OB-*N*(Bu)-cGMP **14** was isolated as a single diastereomer. Additionally, to the *N*-acylated derivative **12**, the NH_2_-unmodified OB-cAMP **15** was prepared. The synthesis was performed analogously to OB-cUMP **10** starting from nucleoside **4** and following synthesis variant a. in which a stepwise addition of the in total applied 2.4 equivalents DCI was performed (Scheme 5).

In summary, despite the need of further yield optimization, five different OB-cNMPs were successfully and reliably synthesized. With regard to the overall similar outcomes of the various preparations, the scope of the reaction can likely be expanded on further nucleosides and/or differently masked phosphoramidites. This makes the chosen approach very flexible, particularly in contrast to the previously reported procedures involving AM esters.

Concluding the syntheses, the functional evaluation of selected OB-cNMPs was performed next.

### 2.2. Functional Evaluation of Selected OB-cNMPs

Ideally, masked precursors of bio-active compounds are biologically inactive and display a stability in physiologic media that on the one hand exceeds their specific activation significantly, and on the other hand guarantees sufficient time for their approximation to their respective target structure. Once activated, the previously inactive compound regains its biologic activity back and should accordingly displays respective effects from target interaction. These demands applied for the prepared OB-cNMPs equally as e.g., for nucleotide prodrugs. Consequently, the chemical stability of the OB-cNMPs under physiologic conditions as well as the efficiency of the enzymatic activation of the OB-mask by an exemplary esterase was evaluated. In complementation, the masked nucleotides were applied in various cell assays to analyze their biologic effects e.g., in the context of Ca^2+^ mobilization and cell activation.

#### 2.2.1. Investigation of Chemical Stability and Enzymatic Activation by PLE

**Figure 3:**
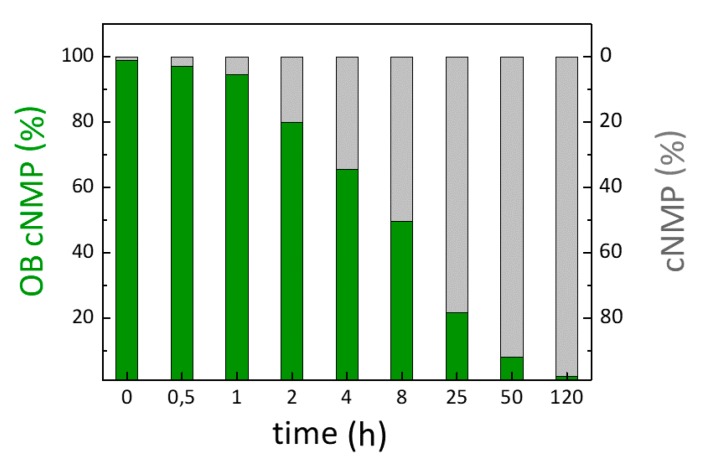
Course of the chemical hydrolysis of OB-*N*(Bu)-cAMP **12** to *N*(Bu)-cAMP **15** given as normalized values for each time point analyzed. Similar hydrolysis courses were measured for OB-cNMPs **10** and **13**.

**Figure 4:**
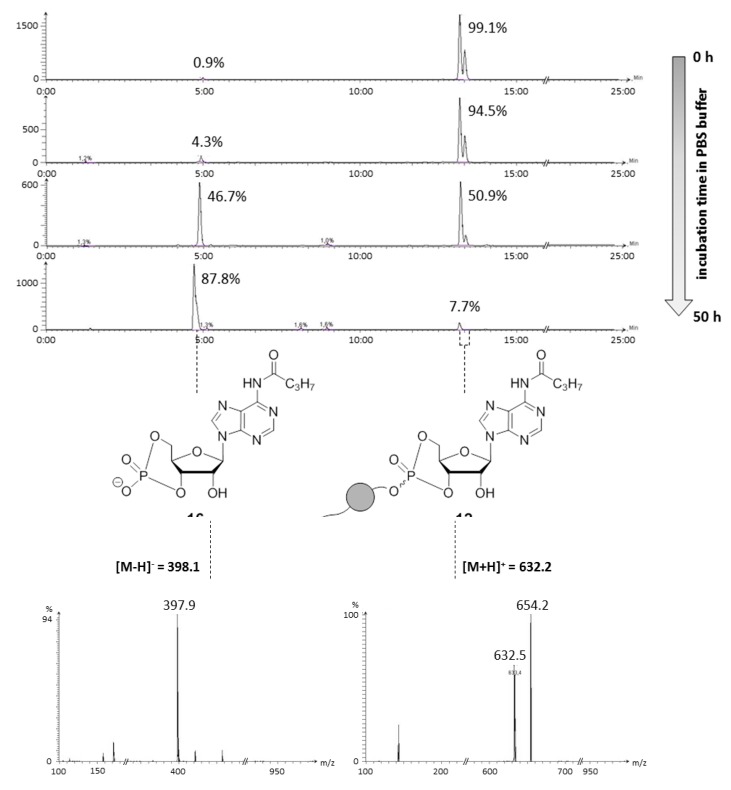
HPLC chromatograms and ESI mass spectra of the chemical hydrolysis of OB-*N*(Bu)-cAMP **12** in PBS (50 mM, pH 7.3) at 0 h, 1 h, 8 h, and 50 h. The two signals of the diastereomers vanished over time while the signal for *N*(Bu)-cAMP **15** increased. The compounds were assigned from the mass spectra recorded at the retention times coinciding with the signals found in the HPLC chromatograms.

Stability determinations for OB-cNMPs **10**, **12**, and **13** (as 5 mM stock sol. in DMSO) were performed in PBS (50 mM, pH 7.3) as a physiologic pH mimic. The compounds (2 mM, final conc. in PBS/DMSO) were incubated for 120 to 200 h at 37 °C. Hydrolysis samples were taken at distinctive times and analyzed via HPLC/MS (Figure 3 and Figure 4). The recorded HPLC chromatograms and ESI mass spectra showed that the OB-cNMPs released only the respective parent cNMP caused by the cleavage of the OB-mask without the formation of further cleavage byproducts (Figure 4).

It was found that after approximately 8 h, 50% of the OB-cNMPs were hydrolyzed (**10**: t_½_ = 8.6 h, **12**: t_½_ = 7.4 h, **13**: t_½_ = 7.5 h) (Figure 3). This half-life implied a stability of the OB-cNMPs that should facilitate convenient setups of cell-based assays and allow experimental set-ups to run, for example, even (pre-)incubation experiments with the masked nucleotides. The hydrolysis behavior of **10**, **12**, and **13** was assessed further, and the relative areas of signals for OB-cNMP and cNMP were determined (in percent). These values were normalized and then averaged for all three hydrolyses under determination of their standard deviation. By this procedure, the individual hydrolysis courses were compared with another since the progress of hydrolyses was expected to be similar as the dissociation of the OB-mask should constitute the determinant process.

The average deviation of the hydrolysis progress for OB-cNMPs **10**, **12**, and **13** at the time points measured was 5% with a single value maximum of 12%. From these calculations, it was deduced that the hydrolysis course was indeed analog for all OB-cNMPs tested and subject to the pace of the cleavage of the masking group and was almost independent of the nucleotide.

**Figure 5:**
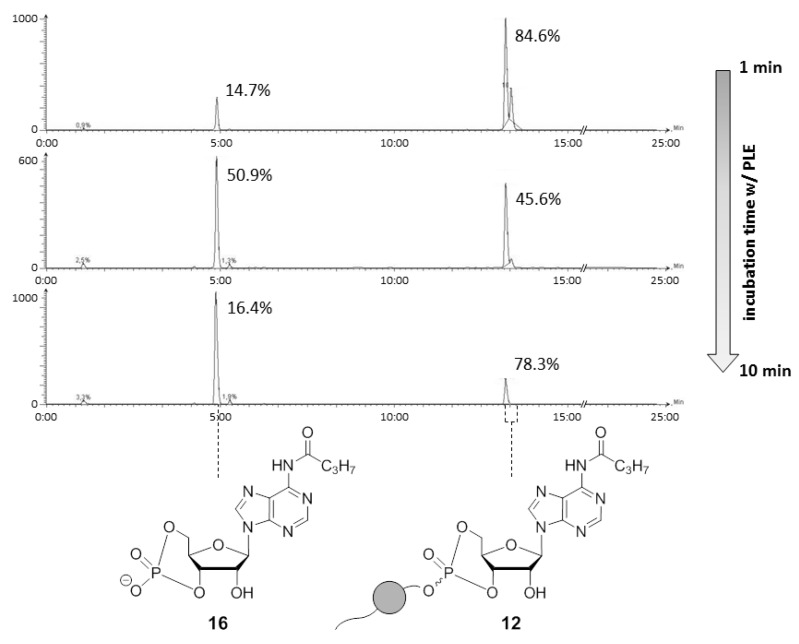
HPLC chromatograms and ESI mass spectra of the enzymatic hydrolysis of OB-*N*(Bu)-cAMP **12** in with PLE (0.05 u/hydrol. sol.) in PBS (50 mM, pH 7.3) at 1, 5, and 10 min. The two signals of the diastereomers vanished over time while the signal for *N*(Bu)-cAMP **15** increased. The compounds were assigned from mass spectra recorded at the retention times coinciding with the signals found in the HPLC chromatograms.

**Figure 6:**
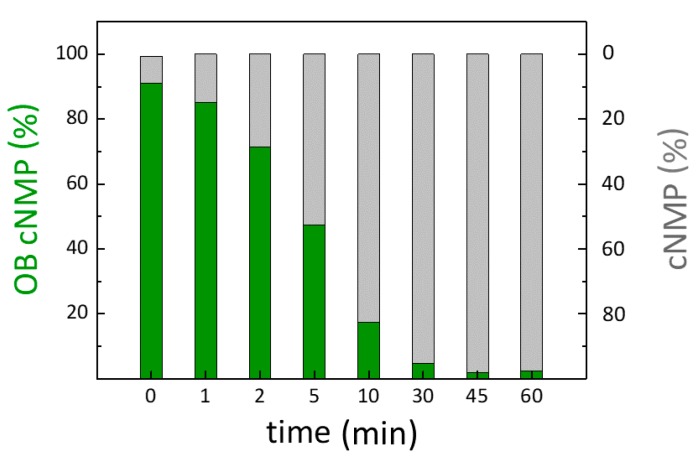
Enzymatic hydrolyses of OB-*N*(Bu)-cAMP **12** to *N*(Bu)-cAMP **15** given as normalized values for each time point analyzed. Similar hydrolysis courses were measured for OB-cNMPs **10** and **13**.

After successfully probing the chemical stability of the OB-cNMPs, their enzymatic activation by pig liver esterase (PLE) as a model esterase was evaluated. The incubations of OB-cNMPs **10**, **12**, and **13** (2 mM final conc. in PBS/DMSO) with PLE were carried out with 0.05 u PLE per hydrolysis sample (V = 20 µL) which enabled good traceability of the enzymatic conversion (Figure 5). Again, no further cleavage products except the corresponding cNMPs were detected. The *N*-butyryl group of **12** and **13** was not cleaved by PLE, even at longer incubation times (up to 60 min), as expected. The chromatograms were processed as described for the chemical hydrolysis to compare the progress of the incubations with PLE (Figure 6).

Half-lives of the studied OB-cNMPs were found to be around 5 min under the applied conditions, which, however, depended significantly on the amount of esterase present. More importantly in this context, the enzymatic activation of OB-cNMPs proceeded even at low PLE concentrations significantly faster than their decomposition in PBS by a factor of approximately 100. In addition, the enzymatic hydrolyses showed analog progression as indicated by a mean deviation of normalized signal areas for OB-cNMPs and cNMPs of 4% with a maximum deviation of 9% for single time point values (Figure 6). This lead again to the conclusion that the enzymatic reaction was almost independent of the type of nucleotide and relied on the OB-mask applied.

In summary, the results of both hydrolysis studies under chemical or enzymatic conditions went well along with the initial criteria as they showed that the stability of the OB-cNMPs was significantly higher than the rate of enzymatic activation. Further, the masked nucleotides proved to be stable enough for application in cell-based assays as their stabilities allow for incubations even over several hours.

Encouraged by the promising hydrolysis properties, the ability of the OB-cNMPs to cross cellular membranes as well as their potential to induce cellular processes was studied next.

#### 2.2.2. Performance of Selected OB-cNMPs in Cell-Based Settings

**Figure 7:**
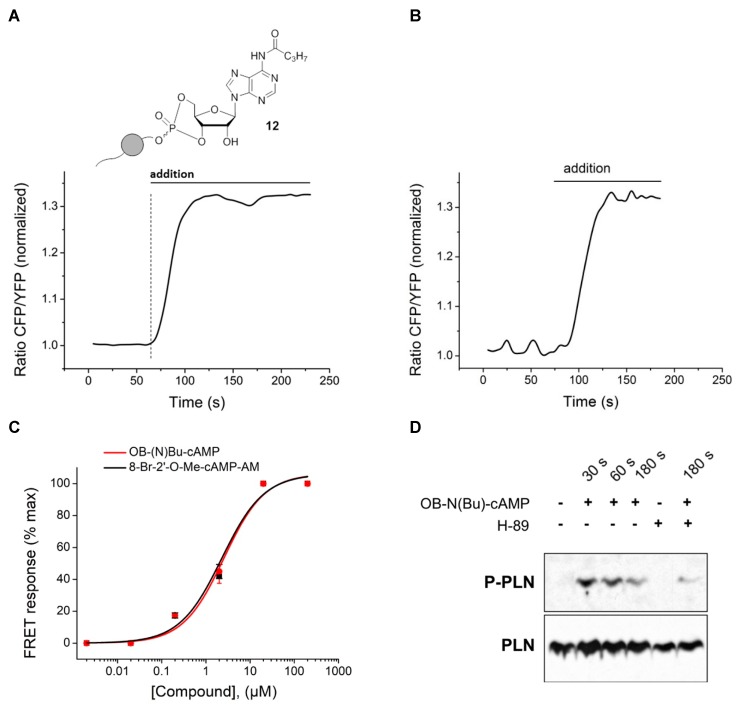
Normalized FRET ratio (between CFP and YFP) over the course of OB-N(Bu)-cAMP **12** addition (20 µM) in (**A**) or of 8-Br-2′-O-Me-cAMP-AM application (20 µM) in (**B**) to mouse cardiomyocytes expressing Epac1-camps biosensor for intracellular cAMP. An instantaneous increase of FRET ratio after addition of **12** to the extracellular medium indicated intracellular release of cAMP and its binding to the FRET biosensor. Representative experiments (n = 5). (**C**) concentration–response dependencies to both analogues show similar potencies (mean ± SEM, n = 5 for each point). (**D**) cardiomyocyte incubation with OB-N(Bu)-cAMP led to rapid phosphorylation of the cAMP dependent protein kinase substrate phospholamban (PLN). Cells were incubated for 30, 90, or 180 s with OB-N(Bu)-cAMP and lysed for immunoblot analysis. Alternatively, cells were preincubated for 20 min with 10 µM of the kinase inhibitor H-89 before 180 s treatment with OB-N(Bu)-cAMP. Representative immunoblots (n = 3).

Primary mouse cardiomyocytes carrying a FRET-sensor with a cAMP binding site were used to examine the membrane-permeability of OB-*N*(Bu)-cAMP **12** in particular. The binding of intracellular cAMP to the FRET sensor is indicated by a decreasing FRET-signal and an increasing fluorescence ratio between cyan-fluorescent protein (CFP) and yellow-fluorescent protein (YFP) [28]. FRET-sensors carrying mouse cardiomyocytes were incubated with OB-*N*(Bu)-cAMP **12** (20 mM DMSO stock sol., 20 µM final conc. in DMSO/buffer, at t ≈ 60 s) (Figure 7A,B). Immediately after addition of **12** to the extracellular medium, the ratio between CFP and YFP started to increase and reached a steady maximum state at circa 50 s (Figure 7A) which was comparable with the previously well-established cell permeable cAMP analogue 8-Br-2′-*O*-Me-cAMP-AM (Figure 7B) (Figure 7A,B) [28]. Likewise, the concentration–response dependencies were largely comparable for both analogues and reached the maximum at 20 µM (Figure 7C). These results imply that OB-*N*(Bu)-cAMP **12** instantaneously crossed the cell membrane and was also rapidly activated by intracellular esterases. Further, the product of the activation process was successfully recognized by the cAMP binding site of the FRET sensor. For the studied substrate, OB-*N*(Bu)-cAMP **12**, this implicated that the *N*^6^-butyryl group was either removed by enzymatic hydrolysis, or that its presence had no detrimental effect on the FRET biosensor interaction. Indeed, as expected from 6-monobutyryl derivatives of cAMP, incubation of cardiomyocytes with **12** led to almost instantaneous of phospholamban phosphorylation by the cAMP dependent protein kinase (Figure 7D). These immunoblots suggest that OB-*N*(Bu)-cAMP might activate phospholamban phosphorylation in a reversible manner. It can be expected from the chemical structure of 6-*N*(Bu)-cAMP that it should be degradable by intramolecular phosphodiesterases, in a way that high activity of these enzymes in cardiomyocytes leads to a gradual decline of cAMP dependent protein kinase substrate phosphorylated over time.

**Figure 8:**
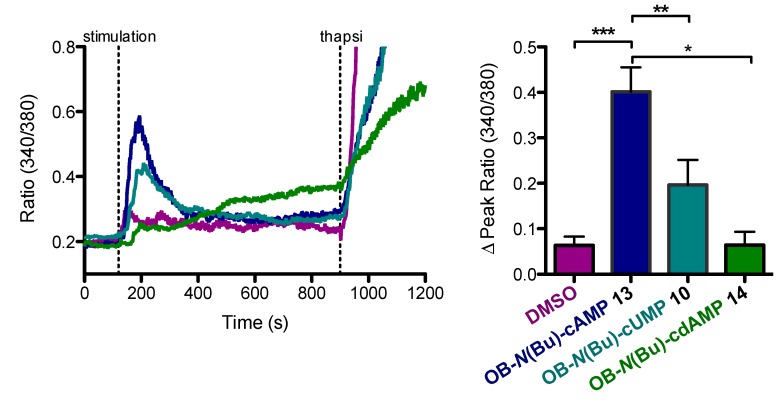
Stimulation of the of Jurkat T cells with OB-cNMPs **10**, **12**, and **13**. Left: Jurkat T cells were stimulated after 120 s with the respective OB-cNMPs (20 µM) or DMSO (as negative control). Furthermore, as positive control Thapsigagarin (1.67 µM) was added after 900 s. Mean signal ratio between 340 nm and 380 nm from single cells are shown (DMSO n = 26; OB cAMP n = 77; OB cUMP n = 37; OB c(dAMP) n = 14). The addition of **12** and **10** resulted in a transient increase of the Ca^2+^ concentration, while no transient increase is visible for **13** or DMSO. Right: Statistical analysis of the mean delta peak for the OB-cNMPs and DMSO (data represent mean ± SEM). The most pronounced effect is measured for **12** and statistically significant differences between are marked by asterisks (* *p* < 0.05, ** *p* < 0.01, *** *p* < 0.001, Kruskal–Wallis Test).

In a second setup, Jurkat T cells were loaded with the Ca^2+^-sensitive fluorescent dye Fura-2. Upon intracellular elevation of Ca^2+^, the absorption ratio between the two excitation wavelengths of Fura-2 at 340 nm and 380 nm increases. This effect correlates directly with the amount of free cytosolic Ca^2+^. Jurkat T cells loaded with Fura-2 were stimulated with OB-cNMPs **10**, **12**, and **13** (20 µM in DMSO, at t ≈ 120 s) added to their extracellular medium (Figure 8). The Fura-2 ratio rose rapidly almost immediately after addition of OB-*N*(Bu)-cAMP **12**, and reached its maximum after approximately 200 s. Then, the Ca^2+^ signal slowly decreased as indicated by the degression of the signal. A similar trend was observed for OB-cUMP **10** but the induced Ca^2+^ signal was significantly reduced compared to **12** (Figure 8). In the case of OB-*N*(Bu)-c(dAMP) **13**, no initial increase of the intracellular Ca^2+^ concentration was measured. However, the ratio seemed to increase slightly over time (Figure 8).

The results confirmed again that the OB-masked cNMPs were able to cross the cell membrane and, importantly, immediately triggered cellular responses. In this context, it was concluded that de-masked cNMPs promoted the observed effects based on the results of the previous hydrolysis studies and the substrate specify Ca^2+^ signaling events display.

The hydrolysis product of **12** acted as would be expected for cAMP, supporting the assumption that the *N*^6^-butyryl group was either removed enzymatically, too, or that its presence did not impede the FRET sensor activation.

Comparison of the measured effects with those evoked by NH_2_-unmodified OB-cAMP **15** and, e.g., further nucleobase derivatives of adenosine or uridine in combination with incubation studies in cell homogenate could help to finalize the analysis and clarify whether the *N*^6^-butyryl group is cleaved or the interacting receptors and binding sites lack selectivity in the corresponding region. Studies along these lines are currently being performed in our laboratories.

In summary, the performed cell assays confirmed excellent membrane-permeability of the selected OB-cNMPs. Further, the cellular effects observed allow the conclusion that the bio-reversible protection at the phosphate was removed rapidly and efficiently. A fast enzymatic activation of the prepared OB-cNMPs was shown analogously in hydrolysis studies using pig liver esterase. The esterase cleaved the OB-mask even at low concentration within very short time, so that similar effects can be expected to proceed in cells. Finally, the observed FRET-sensor binding site interaction and induced Ca^2+^ mobilization proved that biologically active compounds were released out of the masked cNMPs. Moreover, the masked nucleotides triggered processes like those attributed to their parent cNMPs (if existent in nature/identified yet).

## 3. Materials and Methods

All reactions involving water-sensitive reagents were conducted under anhydrous conditions and a dry atmosphere of nitrogen. Reagents were used as purchased from commercial suppliers. Anhydrous *N*,*N*-dimethylformamide (DMF) was purchased and stored over 4 Å molecular sieves. All other anhydrous solvents were purified and dried using a solvent purification system (MB SPS-800 from Braun) and stored over appropriate molecular sieves. Solvents for normal phase (NP) chromatography were distilled prior to use. Acetonitrile was purchased in HPLC grade for reversed phase (RP) chromatography and HPLC. Evaporation of solvents was performed under reduced pressure on a rotary evaporator or using a high vacuum pump. Reactions were monitored via thin layer chromatography (TLC) carried out on pre-coated Macherey-Nagel TLC plates Alugram^®^ Xtra SIL G/UV_254_, and compounds stained with Vanillin (Vanillin (5 g), 1000 mL MeOH/AcOH 9:1, 35 mL H_2_SO_4_) under heating. For automated NP or RP chromatography two flash systems (Interchim Puriflash 430 (Montlucon, France) or Büchi Sepacore^®^ Flash System (Essen, Germany), combined with Macherey-Nagel Chromabond^®^ Flash RS 80 SiOH (NP) or RS40 C_18_ ec (RP) columns (Düren, Germany)) were used. For purifications of phosphordiamidites, a chromatotron (Harrison Research 7924T) with glass plates coated with 2 or 4 mm layers of VWR60 PF_254_ silica gel containing a fluorescent indicator (VWR no. 7749) was used. Analytical RP-High Performance Liquid Chromatography-Mass Spectrometry (RP-HPLC/MS) was performed with an Agilent 1260 Infinity instrument, Waldbronn, Germany (pump G1311B, autosampler G1329B, column compartment G1316A, diode array detector G4221B, column Agilent Poroshell 120 EC-C18, 2.7 mm, 4.6 × 50 mm) coupled with single-quad MS (Advion expression^L^ CMS, Harlow, U.K.). Ultrapure water was generated by a Sartorius Arium^®^ pro unit (Sartopore 0.2 µm, UV, Göttingen, Germany). As elution buffer served a tetra-*n*-butylammonium acetate solution (10 mM, pH 7.2). HPLC/MS runs were performed according to the following method: 0–15 min: water/acetonitrile gradient (2–98% B) with a flow of 0.5 mL/min, 20 °C column temperature and UV detection at 259 nm and 270 nm, MS scans from 150 to 1100 *m*/*z*. Nuclear magnetic resonance (NMR) spectra were recorded at room temperature on Bruker Fourier 300 (300 MHz for ^1^H acquisitions), Bruker AMX 400 (Karlsruhe, Germany) (400 MHz for ^1^H 101 MHz for ^13^C and 152 MHz for ^31^P acquisitions) or Bruker AVIII 600 (Karlsruhe, Germany) (600 MHz for ^1^H and 151 MHz for ^13^C acquisitions) spectrometers in automation mode. All chemical shifts (δ) are given in parts per million (ppm) with the solvent resonance as internal standard. Coupling constants *J* are given in Hertz (Hz). Two-dimensional NMR experiments (HSQC, HMBC) were used for the assignment of quaternary carbons. For mass spectrometric (MS) analysis, spectra were acquired on an Agilent 6224 ESI-TOF spectrometer (Waldbronn, Germany) in positive and negative mode as required. Infrared spectroscopy (IR) was carried out with a Bruker Alpha P FT-IR (Bremen, Germany) in attenuated total reflection (ATR) mode at room temperature ranging from 400 cm^−1^ to 4000 cm^−1^. For FRET measurements, primary mouse ventricular cardiomyocytes were isolated from Epac1-camps biosensor expressing transgenic mice [29] as described [28] and plated onto laminin coated glass cover slides. Measurements were performed 1–2 h after plating using a Nikon Ti microscope based FRET imaging system containing pE-100 440 nm light source (CoolLED), DV2 Dual View and ORCA-03G charge-coupled device camera (Hamamatsu, Japan), and analyzed as described [28]. Cells were kept in a buffer containing 144 mM NaCl, 5.4 mM KCl, 1 mM MgSO_4_, 1 mM CaCl_2_, 10 mM Hepes (pH 7.3) and stimulated with OB-cNMPs or 8-Br-2′-O-Me-cAMP-AM (purchased from BIOLOG Life Science Institute, Bremen, Germany) dissolved 1:1000 in the same buffer from a freshly made 20 mM DMSO stock solution. For Ca^2+^ mobilization assays, Jurkat T cells were incubated with the membrane-permeable AM ester of the Ca^2+^ dye Fura-2 (4 µM, Calbiochem). Therefore, about 2 × 10^6^ cells were centrifuged at 500 g for 5 min and resuspended in 1 mL of freshly supplemented RPMI medium containing Fura-2 AM. Cells were incubated for 30 min at 37 °C. After centrifugation, cells were washed and resuspended in Ca^2+^ buffer (140 mM NaCl, 5 mM KCl, 1 mM MgSO_4_, 1 mM CaCl_2_, 20 mM Hepes (pH 7.4), 1 mM NaH_2_PO_4_, 5 mM glucose) and kept for 20 min at rt for de-esterification. Cells were added on prepared coverslips and allowed to adhere before measurement. Slides were mounted onto a Leica IRBE microscope (100-fold magnification) and after 120 s the respective OB-cNMPs (20 µM) or DMSO (as control) were added. As positive control, Thapsigagarin (1.67 µM, Calbiochem) was added after 900 s. A Sutter DG-4 was used as a light source, and frames were acquired with an electron-multiplying charge-coupled device camera (C9100-13, Hamamatsu). Images (512 × 512 pixels) were acquired in 16-bit mode with the following filter sets (AHF Analysentechnik) (excitation (ex), beam splitter (bs), and emission (em), all in nanometers): Fura-2 (ex, HC 340/26, HC 387/11; bs, 400DCLP; em, 510/84).

For immunoblot analysis, freshly isolated cardiomyocytes sedimented in stop buffer without serum were preincubated for 30 min either with vehicle or with 10 µM H-89 (Sigma). Next, 20 µM of OB-N(Bu)-cAMP were added to various time points (30, 90, and 180 s), after which cells were lysed and processed for immunoblot analysis. Homogenized proteins were quantified using BCA Protein Assay (Pierce). Samples (20 µg protein per lane) were boiled at 95 °C for 5 min to disrupt phospholamban pentamers, loaded onto 15% SDS–polyacrylamide gel and immunoblotting using the anti-P-Ser16 phospholamban (Badrilla 1:5.000), and total anti-PLN A-1 antibodies (Badrilla).

### 3.1. General Procedures

GP I: *N*-butanoylation of nucleosides via transient TMS-protection:

The respective nucleoside (adenosine, guanosine, or 2′-deoxyadenosine) was co-evaporated three times and then dissolved in anhydrous pyridine (2–5 mL/mmol), and diluted either with the same volume of THF or double the volume of CH_2_Cl_2_. At 0 °C, TMSCl (2.1–9.0 equiv.) was added. The reaction mixture was allowed to warm up to rt and stirred for 5–18 h. Successively, butyryl chloride (1.1 equiv.) was added slowly and the reaction mixture stirred for another 6 h at rt. Cleavage of TMS ethers was promoted by the addition of either 1 M HCl (0.5 mL/mmol) under vigorous stirring for 5 min, or methanol (2–5 mL/mmol) and stirring at rt for further 12 h. The reaction was terminated by removal of all volatile components under high vacuum. The crude residue was co-evaporated with toluene and CH_2_Cl_2_ several times, and then taken up in acetonitrile/demin. water. Purification was performed by means of automated RP flash column chromatography on C_18_ modified silica gel with an acetonitrile gradient in water (0% to 100%).

GP II: 3′,5′-Phosphorylation of nucleosides to their OB-masked cNMP analogues:

Under an atmosphere of nitrogen, the respective nucleoside was dissolved in DMF/CH_3_CN (25 mL/mmol). In a separate flask, bis(*N*,*N*-di*iso*propylamino)-4-octanoyloxybenzyl phosphoramidite (1 equiv.) was dissolved in acetonitrile (25 mL/mmol total volume). The phosphor diamidite solution and DCI (0.25 M in CH_3_CN, 1.3–1.5 equiv.) were added slowly and dropwise in five to ten portions to the nucleoside solution. The addition of more DCI (0.25 M in CH_3_CN, 1.2–1.5 equiv.) or 5-(benzylthio)-1*H*-tetrazole (BTT, 0.3 M in CH_3_CN, 1.3 equiv.) followed, and the reaction mixture was stirred 1 h further. Then, *t*BuOOH (5.5 M in *n*-decane, 1.5 equiv.) was added and the solution stirred for 10 min more. Successively, all volatile components were removed in vacuum, and the obtained residue was taken up in CH_3_CN/demin. water and purified by means of an automated RP flash column chromatography on C_18_ modified silica gel with an CH_3_CN gradient in water (0% to 100%).

### 3.2. Syntheses

#### 3.2.1. Synthesis of bis(*N*,*N*-di*iso*propylamino)-4-octanoyloxybenzyl phosphordiamidite **3**

1.00 g (3.75 mmol) bis(*N*,*N*-di*iso*propylamino)chlorophosphine **1** were dissolved in 15 mL anhydrous THF. In a separate flask, 0.68 mL (4.87 mmol, 1.3 equiv.) NEt_3_ and 0.94 g (3.75 mmol, 1 equiv.) 4-(hydroxymethyl)phenyloctanoate **2** were mixed with 7 mL anhydrous THF, and the mixture was added dropwise to the chlorophosphine. The reaction mixture was stirred at rt for 20 h, then filtrated and the filtrate concentrated to dryness in vacuum. The remaining residue was purified by NP chromatography on silica gel with PE/TEA 98:2 as eluents, and the desired product obtained as colorless syrup.

Yield: 1.28 g (2.67 mmol, 71%).

^1^H-NMR (600 MHz, chloroform-d): δ [ppm] = 7.36 (d, ^2^*J*_H,H_ = 8.2 Hz, 2H), 7.14–6.95 (m, 2H), 4.63 (d, ^2^*J*_H,H_ = 7.2 Hz, 2H), 3.66–3.51 (m, 4H), 2.54 (t, ^2,3^*J*_H,H_ = 7.5 Hz, 2H), 1.75 (p, ^2,3^*J*_H,H_ = 7.4 Hz, 2H), 1.51–1.23 (m, 8 H), 1.21 (d, ^2^*J*_H,H_ = 2.7 Hz, 12H), 1.20 (d, ^2^*J*_H,H_ = 2.8 Hz, 12H) 0.88 (t, ^2,3^*J*_H,H_ = 7.3 Hz, 3H); ^13^C{^1^H}-NMR (151 MHz, chloroform-d): δ [ppm] = 172.6, 149.7, 138.2, 127.9, 121.5, 65.8, 44.69, 44.56, 34.6, 31.8, 29.2, 29.1, 25.1, 24.8, 24.7, 24.1, 24.0, 22.75, 14.22; ^31^P{^1^H}-NMR (162 MHz, chloroform-d): δ [ppm] = 123.5. IR (ATR): ṽ in [cm^−1^] = 2963.3, 2927.8, 2861.4, 2079.0, 2025.5, 1761.3, 1607.8, 1507.4, 1457.5, 1416.5, 1390.1, 1361.6, 1300.3, 1194.2, 1184.8, 1162.9, 1140.3, 1116.2, 1045.2, 1016.9, 952.7, 916.5, 866.3, 779.6, 748.7, 706.7, 642.7, 566.1, 527.6. MS (MALDI): *m*/*z* [M-H] calcd. for C_27_H_48_N_2_O_3_P^-^: 479.340, found: 479.245.

#### 3.2.2. Synthesis of 6-*N*-butanoyl-adenosine **7**

In accordance with GP I, 1.40 g (5.26 mmol) adenosine **4** were dissolve in 34 mL pyridine/THF 1:1 and converted with 2.11 mL (16.5 mmol, 3.2 equiv.) TMSCl and 0.60 mL (5.78 mmol, 1.1 equiv.) butyryl chloride. After 6 h stirring at rt, 2.5 mL 1 M HCl (aq.) was added to cleave the TMS ethers, and after 5 min, all volatile components were removed under vacuum. Upon final purification of the crude product via automated RP flash column chromatography on C_18_ modified silica gel with an CH_3_CN gradient in water (0% to 100%), the product was obtained as colorless powder.

Yield: 1.09 g (3.22 mmol, 61%).

^1^H-NMR (500 MHz, DMSO-d_6_): δ [ppm] = 10.63 (s, 1H), 8.69 (s, 1H), 8.65 (s, 1H), 6.01 (d, ^3^*J*_H,H_ = 5.8 Hz, 1H), 5.53 (d, ^3^*J*_H,H_ = 5.8 Hz, 1H), 5.23 (d, ^3^*J*_H,H_ = 4.8 Hz, 1H), 5.12 (t, ^3^*J*_H,H_ = 5.6 Hz, 1H), 4.63 (q, ^3^*J*_H,H_ = 5.4 Hz, 1H), 4.19 (q, ^3^*J*_H,H_ = 4.3 Hz, 1H), 3.98 (q, ^3^*J*_H,H_ = 3.9 Hz, 1H), 3.76–3.64 (m, 1H), 3.58 (ddd, ^2^*J*_H,H_ = 11.9 Hz, ^3^*J*_H,H_ = 6.1 Hz, ^3^*J*_H,H_ = 4.0 Hz, 1H), 2.55 (t, ^2,3^*J*_H,H_ = 7.3 Hz, 2H), 1.63 (h, ^2,3^*J*_H,H_ = 7.4 Hz, 2H), 0.94 (t, ^2,3^*J*_H,H_ = 7.4 Hz, 3H); ^13^C-NMR (126 MHz, DMSO-d_6_): δ [ppm] = 171.5, 151.7, 151.6, 149.7, 142.7, 123.9, 87.6, 85.7, 73.7, 70.3, 61.3, 38.0, 18.2, 13.5. IR (ATR): ṽ in [cm^−1^] = 3273.2, 2963.3, 2934.0, 2875.0, 1716.3, 1685.0, 1613.3, 1587.0, 1521.9, 1460.3, 1409.0, 1356.6, 1327.0, 1224.3, 1124.1, 1082.2, 1056.7, 984.6, 902.4, 866.3, 799.6, 745.0, 704.7, 643.7, 547.3. MS (ESI-HR): *m*/*z* [M + H]^+^ calcd. for C_14_H_20_N_5_O_5_^+^: 338.1459, found: 338.1469.

#### 3.2.3. Synthesis of 6-*N*-butanoyl-2′-deoxyadenosine **8**

Following GP I, 1.14 g (4.24 mmol) 2′-deoxyadenosine **5** were dissolve in 24 mL pyridine/CH_2_Cl_2_ 1:2. At 0 °C, 1.13 mL (8.91 mmol, 2.1 equiv.) TMSCl were added, and the reaction mixture was stirred for 18 h at rt. Successively, 0.48 mL (4.67 mmol, 1.1 equiv.) butyryl chloride were added. After further 3 h stirring at rt, the TMS ethers were removed by addition of 8 mL CH_3_OH at 0 °C. After further 5 h at rt, the reaction was terminated by removing all volatile components under vacuum. The crude product was taken up in water containing little amount of CH_3_CN and purified via automated RP flash column chromatography on C_18_ modified silica gel with an CH_3_CN gradient in water (0% to 100%) to afford the desired product as colorless powder.

Yield: 0.43 g (1.35 mmol, 32%).

^1^H-NMR (400 MHz, methanol-d_4_): δ [ppm] = 8.66 (s, 1H), 8.62 (s, 1H), 6.61–6.52 (m, 1 H), 4.64 (dt, ^3^*J*_H,H_ = 6.1 Hz, ^3^*J*_H,H_ = 3.1 Hz, 1H), 4.10 (q, ^3^*J*_H,H_ = 3.4 Hz, 1H), 3.88 (dd, ^2^*J*_H,H_ = 12.2 Hz, ^3^*J*_H,H_ = 3.4 Hz, 1H), 3.79 (dd, ^2^*J*_H,H_ = 12.2 Hz, ^3^*J*_H,H_ = 3.9 Hz, 1H), 2.88 (ddd, ^2^*J*_H,H_ = 13.4 Hz, ^3^*J*_H,H_ = 7.4 Hz, ^3^*J*_H,H_ = 6.0 Hz, 1H), 2.67 (t, ^2,3^*J*_H,H_ = 7.4 Hz, 2H), 2.51 (ddd, ^2^*J*_H,H_ = 13.5 Hz, ^3^*J*_H,H_ = 6.2 Hz, ^3^*J*_H,H_ = 3.3 Hz, 1H), 1.82 (h, ^2,3^*J*_H,H_ = 7.4 Hz, 2H), 1.08 (t, ^2,3^*J*_H,H_ = 7.4 Hz, 3H); ^13^C{^1^H}-NMR (101 MHz, methanol-d_4_): δ [ppm] = 174.4, 152.9, 150.7, 144.3, 123.2, 89.7, 86.7, 72.7, 63.4, 41.5, 39.9, 19.6, 14.0. IR (ATR): ṽ in [cm^−1^] = 3336.8, 2964.6, 2933.1, 2875.3, 2592.1, 2330.9, 1682.2, 1612.5.1585.9, 1522.5, 1459.6, 1402.8, 1354.8, 1329.2, 1223.8, 1093.3, 1058.5, 993.8, 941.1, 867.1, 799.6, 749.6, 984.8, 644.4, 585.3, 561.2, 542.4, 527.4, 509.6, 464.7. MS (ESI-HR): *m*/*z* [M + H]^+^ calcd. for C_14_H_20_N_5_O_4_^+^: 322.1510, found: 322.1521.

#### 3.2.4. Synthesis of 2-*N*-butanoyl-guanosine **9**

According to GP I, 1.55 g (5.48 mmol) guanosine **6** were co-evaporated with pyridine three times and then dissolved in 81 mL pyridine/CH_2_Cl_2_ 1:2. At 0 °C, 6.28 mL (49.4 mmol, 9 equiv.) TMSCl were added, and the reaction mixture was stirred for 4 h at rt. Successively, 0.62 mL (6.03 mmol, 1.1 equiv.) butyryl chloride were added. After 3 h stirring at rt, the cleavage of the TMS ethers was induced by addition of 27 mL CH_3_OH, and after further 12 h at rt, the reaction was terminated and all volatile components were removed under vacuum. The crude residue was taken up in water containing little amount of CH_3_CN and finally purified via automated RP flash column chromatography on C_18_ modified silica gel with an CH_3_CN gradient in water (0% to 100%) to afford the desired product as colorless powder.

Yield: 0.92 g (2.61 mmol, 48%).

^1^H-NMR (400 MHz, DMSO-d_6_): δ [ppm] = 12.07 (bs, 1H), 11.71 (bs, 1H), 8.26 (s, 1 H), 5.80 (d, ^3^*J*_H,H_ = 5.7 Hz, 1H), 5.47 (d, ^3^*J*_H,H_ = 5.7 Hz, 1H), 5.17 (d, ^3^*J*_H,H_ = 4.5 Hz, 1H), 5.03 (t, ^3^*J*_H,H_ = 5.4 Hz, 1H), 4.43 (d, ^3^*J*_H,H_ = 5.2 Hz, 1H), 4.20–4.05 (m, 1H), 3.90 (q, *J* = 3.9 Hz, 1H), 3.64 (dt, ^2^*J*_H,H_ = 11.9 Hz, ^3^*J*_H,H_ = 4.8 Hz, 1H), 3.55 (dt, ^2^*J*_H,H_ = 11.9 Hz, ^3^*J*_H,H_ = 4.7 Hz, 1H), 2.45 (t, ^2,3^*J*_H,H_ = 7.3 Hz, 2H), 1.62 (h, ^2,3^*J*_H,H_ = 7.4 Hz, 2H), 0.92 (t, ^2,3^*J*_H,H_ = 7.5 Hz, 3H); ^13^C{^1^H}-NMR (101 MHz, DMSO-d_6_): δ [ppm] = 176.2, 154.8, 148.8, 148.0, 137.6, 120.1, 86.6, 85.3, 73.9, 61.1, 37.8, 17.9, 13.4. IR (ATR): ṽ in [cm^−1^] = 3364.4, 3279.5, 2968.3, 2941.1, 1680.2, 1608.8, 1564.3, 1554.1, 1536.0, 1481.4, 1469.2, 1449.9, 1402.5, 1251.7, 1204.2, 1179.4, 1127.9, 1089.0, 1060.2, 993.3, 976.4, 901.1, 863.3, 818.6, 801.0, 762.7, 737.4, 717.0, 680.4, 643.7, 607.0, 589.1, 562.3, 510.2, 484.2. MS (ESI-HR): *m*/*z* [M + H]^+^ calcd. for C_14_H_19_N_5_O_6_^+^: 354.1408, found: 354.1400.

#### 3.2.5. Synthesis of Uridine-3′,5′-(4-octanoyloxybenzyl)cyclophosphate **10**

According to GP II, 26 mg (0.11 mmol) uridine **10** were dissolved in 2 mL DMF and reacted with 57 mg (0.12 mmol, 1.1 equiv.) OB-PA_2_ **3**, dissolved in 2.5 mL CH_3_CN, in the presence of 0.54 mL (0.13 mmol, 1.3 equiv.) DCI (0.25 M in CH_3_CN) and 0.45 mL (0.13 mmol, 1.3 equiv.) BTT (0.3 M in CH_3_CN). The addition of 32 µL (0.16 mmol, 1.5 equiv.) *t*BuOOH (5.5 M in *n*-decane) followed successively. After purification by automated RP flash column chromatography on C_18_ modified silica gel with a CH_3_CN gradient in water (0% to 100%) and lyophilization, the product was obtained as colorless cotton and in two fractions containing each of the stereoisomers.

Yield: 11 mg (0.02 mmol, 19%).

^1^H-NMR (400 MHz, methanol-d_4_): δ [ppm] = 7.66–7.51 (m, 2H), 7.47 (d, ^3^*J*_H,H_ = 8.1 Hz, 1H), 7.22–7.08 (m, 2H), 5.69 (d, ^3^*J*_H,H_ = 8.0 Hz, 1H), 5.64 (d, ^3^*J*_H,H_ = 0.9 Hz, 1H), 5.24–5.14 (m, 2H), 4.64 (ddd, ^3^*J*_H,P_ = 22.5 Hz, ^2^*J*_H,H_ = 9.3 Hz, ^3^*J*_H,H_ = 4.6 Hz, 1H), 4.39 (ddd, ^3^*J*_H,H_ = 10.2, ^2^*J*_H,H_ = 9.4, ^3^*J*_H,H_ = 0.8 Hz, 1H), 4.35–4.25 (m, 2H), 4.25–4.10 (m, 1H), 2.58 (t, ^2,3^*J*_H,H_ = 7.4 Hz, 2H), 1.73 (p, ^2,3^*J*_H,H_ = 7.4 Hz, 2H), 1.53–1.22 (m, 8H), 1.05–0.82 (m, 3H)

and

7.63 (d, ^3^*J*_H,H_ = 8.1 Hz, 1H), 7.53–7.47 (m, 2H), 7.19–7.10 (m, 2H), 5.73 (d, ^3^*J*_H,H_ = 1.2 Hz, 1H), 5.71 (d, ^3^*J*_H,H_ = 8.1 Hz, 1H), 5.19 (d, ^2^*J*_H,H_ = 8.7 Hz, 2H), 4.81 (ddd, ^3^*J*_H,P_ = 9.8 Hz, ^3^*J*_H,H_ = 5.1 Hz, ^3^*J*_H,H_ = 1.0 Hz, 1H), 4.67 (ddd, ^3^*J*_H,P_ = 14.2 Hz, ^2^*J*_H,H_ = 9.2 Hz, ^3^*J*_H,H_ = 5.5 Hz, 1H), 4.62–4.47 (m, 2H), 4.42 (td, ^2^*J*_H,H_ = 10.1, ^3^*J*_H,H_ = 5.5 Hz, 1H), 2.60 (t, ^2,3^*J*_H,H_ 7.4 Hz, 2H), 1.74 (p, ^2,3^*J*_H,H_ 7.4 Hz, 2H), 1.50–1.26 (m, 8H), 1.02–0.84 (m, 3H); ^13^C{^1^H}-NMR (101 MHz, methanol-d_4_): δ [ppm] = 173.8, 165.9, 151.5, 150.1, 143.7, 134.3, 131.2, 123.3, 103.0, 97.5, 80.1, 72.4, 71.5, 71.2, 70.3, 35.0, 32.9, 30.2, 30.1, 25.0, 23.7, 14.4

and

172.8, 164.9, 151.7, 150.5, 143.5, 133.0, 130.2, 122.8, 103.1, 97.7, 79.3, 72.4, 71.2, 71.1, 70.6, 35.0, 32.8, 30.2, 30.1, 26.0, 23.7, 14.4; ^31^P{^1^H}-NMR (162 MHz, methanol-d_4_): δ [ppm] = −3.93, −5.01. MS (ESI-HR): *m*/*z* [M + Na]^+^ calcd. for C_24_H_31_N_2_O_10_PNa^+^: 561.1609, found: 561.1445.

#### 3.2.6. Synthesis of 6-*N*-butanoyl-adenosine-3′,5′-(4-octanoyloxybenzyl)cyclophosphate **12**

According to GP II, 48 mg (0.14 mmol) 6-*N*-butanoyl-adenosine **7** were dissolved in 4 mL DMF and treated with a solution of 76 mg (0.16 mmol, 1.1 equiv.) OB-PA_2_ **3** in 4 mL CH_3_CN and 0.97 mL (0.24 mmol, 1.7 equiv.) DCI (0.25 M in CH_3_CN) as well as 0.62 mL (0.19 mmol, 1.3 equiv.) BTT (0.3 M in CH_3_CN). Then, 43 µL (0.21 mmol, 1.5 equiv.) *t*BuOOH (5.5 M in *n*-decane) were added. After purification by automated RP flash chromatography on C_18_ modified silica with a CH_3_CN gradient in water (0% to 100%), the product was obtained as colorless cotton and mixture of two diastereomers.

Yield: 13 g (0.02 mmol, 14%).

^1^H-NMR (400 MHz, methanol-d_4_): δ [ppm] = 8.68, 8.58 (2× s, 2H), 8.49, 8.38 (2× s, 2H), 7.62–7.56, 7.55–7.49 (2× m, 4H), 7.17–7.12, 7.13–7.06 (2× m, 4H), 6.20, 6.12 (2× s, 2H), 5.47 (dd, ^3^*J*_H,P_ = 9.1 Hz, ^3^*J*_H,H_ = 5.1 Hz, 1H) 5.23 (2× d, ^2^*J*_H,H_ = 10.4 Hz & 8.1 Hz, 4H), 5.05 (ddd, ^3^*J*_H,P_ = 9.6 Hz, ^3^*J*_H,H_ = 5.1 Hz, ^3^*J*_H,H_ = 1.6 Hz, 1H), 4.89–4.86 (m, 1H), 4.73–4.34 (m, 7H), 2.71–2.55 (m, 6H,), 2.51 (t, ^2,3^*J*_H,H_ = 7.4 Hz, 2H), 1.86–1.70 (m, 6H), 1.68–1.57 (m, 2H), 1.53–1.25 (m, 16H), 1.05 (2× t, ^2,3^*J*_H,H_ = 7.4 Hz, 6H), 0.98–0.95 (m, 6H); ^13^C{^1^H}-NMR (151 MHz, methanol-d_4_): δ [ppm] = 173.8, 173.6, 152.8, 152.4, 150.8, 143.1, 133.3, 133.2, 130.7, 130.6, 124.5, 123.1, 123.0, 88.8, 81.7, 80.4, 69.3, 69.2, 68.5, 59.8, 39.9, 34.5, 31.8, 29.2, 29.1, 25.0, 22.8, 18.5,14.2, 13.9; ^31^P{^1^H}-NMR (162 MHz, methanol-d_4_): δ [ppm] = −3.80, −4.90. MS (ESI-HR): *m*/*z* [M + H]^+^ calcd. for C_29_H_39_N_5_O_9_P^+^: 632.2848, found: 632.2848.

#### 3.2.7. Synthesis of 6-*N*-butanoyl-2′-deoxyadenosine-3′,5′-(4-octanoyloxybenzyl)cyclophosphate **13**

According to GP II, 50 mg (0.15 mmol) 6-*N*-butanoyl-2’-deoxyadenosine **8** were dissolved in 4 mL DMF and reacted with 82 mg (0.17 mmol, 1.1 equiv.) OB-PA_2_ **3**, dissolved in 4.3 mL CH_3_CN, in the presence of 1.60 mL (0.40 mmol, 2.6 equiv.) DCI (0.25 M in CH_3_CN) in total and 50 µL (0.25 mmol, 1.6 equiv.) *t*BuOOH (5.5 M in *n*-decane). After purification by automated RP flash column chromatography on C_18_ modified silica gel with a CH_3_CN gradient in water (0% to 100%) and lyophilization, the product was obtained as colorless cotton and mixture of two diastereomers.

Yield: 12 mg (0.02 mmol, 13%).

^1^H-NMR (400 MHz, methanol-d_4_): δ [ppm] = 8.69 (s, 1H), 8.61 (s, 1H), 8.47 (s, 1H), 8.37 (s, 1H), 7.65–7.57, 7.55–7.48 (2× m, 2H), 7.19–7.10 (m, 4H), 6.59 (dd, ^3^*J*_H,H_ = 9.0 Hz, ^3^*J*_H,H_ = 2.0 Hz, 1H), 6.55 (dd, ^3^*J*_H,H_ = 6.7 Hz, ^3^*J*_H,H_ = 4.0 Hz, 1H), 5.64 (q, ^3^*J*_H,H/P_ = 9.2 Hz, 1H), 5.33 (q, ^3^*J*_H,H/P_ = 9.2 Hz, 1H), 5.23 (2× d, ^2^*J*_H,H_ = 13.1 Hz & ^3^*J*_H,H_ = 12.9 Hz, 4H), 4.70–4.40 (m, 4H), 4.22 (td, ^3^*J*_H,H_ = 9.9 Hz, ^3^*J*_H,H_ = 5.6 Hz, 1H), 4.08 (td, ^3^*J*_H,H_ = 9.9 Hz, ^3^*J*_H,H_ = 4.7 Hz, 1H), 2.98 (ddd, ^2^*J*_H,H_ = 13.1 Hz, ^3^*J*_H,H_ = 7.7 Hz, ^3^*J*_H,H_ = 1.9 Hz, 1H), 2.83–2.70 (m, 3H), 2.69–2.57 (m, 6H), 2.54 (t, ^2,3^*J*_H,H_ = 7.4 Hz, 2H), 1.90–1.70 (m, 6H), 1.66 (q, ^2,3^*J*_H,H_ = 7.3 Hz, 2H), 1.51–1.26 (m, 16H), 1.06 (2 x t, ^2,3^*J*_H,H_ = 7.4, 6H), 0.98–0.86 (m, 6H); ^13^C{^1^H}-NMR (101 MHz, methanol-d_4_): δ [ppm] = 174.5, 174.4, 173.8, 173.6, 153.4, 153.2, 152.8, 152.7, 152.4, 152.3, 150.8, 150.7, 144.6, 144.5, 134.4, 134.3, 131.1, 130.7, 123.2, 123.1, 122.6, 85.8, 85.6, 79.3, 79.2, 75.7, 75.6, 71.7, 71.6, 71.4, 71.3, 40.0, 39.9, 35.0, 34.9, 32.8, 30.1, 30.0, 25.9, 25.9, 23.7, 19.7, 19.6, 14.4, 14.1, 14.0; ^31^P{^1^H}-NMR (162 MHz, methanol-d_4_): δ [ppm] = −4.14, −5.50. MS (ESI-HR): *m*/*z* [M + H]^+^ calcd. for C_29_H_39_N_5_O_8_P^+^: 616.2531, found: 616.2534.

#### 3.2.8. Synthesis of 2-*N*-butanoyl-guanosine-3′,5′-(4-octanoyloxybenzyl)cyclophosphate **14**

In accordance with GP II, 52 mg (0.15 mmol) 6-*N*-butanoyl-guanosine **9** were dissolved in 4 mL DMF and treated with a solution of 77 mg (0.16 mmol, 1.1 equiv.) OB-PA_2_ **3** in 4 mL CH_3_CN, 1.75 mL (0.44 mmol, 3 equiv.) DCI (0.25 M in CH_3_CN) in total and finally, 44 µL (0.21 mmol, 1.5 equiv.) *t*BuOOH (5.5 M in *n*-decane). After purification by automated RP flash column chromatography on C_18_ modified silica gel with a CH_3_CN gradient in water (0% to 100%) and lyophilization, the product was obtained as colorless cotton and a single diastereomer.

Yield: 4 mg (5.6 µmol, 4%).

^1^H-NMR (400 MHz, methanol-d_4_): δ [ppm] = 7.92 (s, 1H), 7.56 (d, ^3^*J*_H,H_ = 8.2 Hz, 2H), 7.07 (d, ^3^*J*_H,H_ = 8.3 Hz, 2H), 6.01 (s, 1H), 5.23 (d, ^2^*J*_H,H_ = 11.6 Hz, 2H), 4.69 (ddd, ^3^*J*_H,H_ = 22.2 Hz, ^3^*J*_H,P_ = 9.4 Hz, ^3^*J*_H,H_ = 4.8 Hz, 1H), 4.55 (t, ^3^*J*_H,H_ = 10.0 Hz, 1H), 4.35 (dd, ^3^*J*_H,H_ = 10.0 Hz, ^3^*J*_H,H_ = 4.8 Hz, 1H), 4.32 (d, ^3^*J*_H,H_ = 4.7 Hz, 1H), 4.18 (dd, ^3^*J*_H,P_ = 9.9 Hz, ^3^*J*_H,H_ = 4.7 Hz, 1H), 2.51 (t, ^2,3^*J*_H,H_ = 7.4 Hz, 2H), 2.46 (t, ^2,3^*J*_H,H_ = 7.1 Hz, 2H), 1.74 (h, ^2,3^*J*_H,H_ = 7.4 Hz, 2H), 1.62 (t, ^2,3^*J*_H,H_ = 7.1 Hz, 2H), 1.49–1.25 (m, 8H,), 1.02 (t, ^2,3^*J*_H,H_ = 7.4 Hz, 3H), 0.98–0.86 (m, 3H); ^13^C{^1^H}-NMR (101 MHz, methanol-d_4_): δ [ppm] = 177.7, 173.6, 152.2, 150.0, 149.8, 137.5, 134.4, 131.4, 123.3, 120.2, 93.4, 80.3, 73.3, 71.6, 71.5, 70.5, 39.4, 34.8, 32.9, 30.1, 25.8, 23.7, 19.3, 14.4, 13.9; ^31^P{^1^H}-NMR (162 MHz, methanol-d_4_): δ [ppm] = −4.93. MS (ESI-HR): *m*/*z* [M + H]^+^ calcd. for C_29_H_39_N_5_O_10_P^+^: 648.2429, found: 648.2720.

#### 3.2.9. Synthesis of adenosine-3′,5′-(4-octanoyloxybenzyl)cyclophosphate **15**

Following GP II, 21 mg (78 µmol) adenosine were dissolved in 2 mL DMF and treated with a solution of 41 mg (85 µmol, 1.1 equiv.) (OB)PA_2_ **101** in 2 mL CH_3_CN as well as 0.74 mL (0.19 mmol, 2.4 equiv.) DCI (0.25 M in CH_3_CN). Lastly, 23 µL (0.12 mmol, 1.5 equiv.) *t*BuOOH (5.5 M in *n*-decane) were added for oxidation. After purification by automated RP flash column chromatography on C_18_ modified silica gel with a CH_3_CN gradient in water (0% to 100%) and lyophilization, the product was obtained as a colorless cotton and mixture of two diastereomers.

Yield: 5 mg (10 µmol, 12%).

^1^H-NMR (400 MHz, acetonitrile-d_3_): δ [ppm] = 8.19 (s, 1H), 8.09 (s, 1 H), 7.93 (s, 1H), 7.88 (s, 1H), 7.55–7.46 (m, 2H), 7.45–7.41 (m, 2H), 7.12–7.02 (m, 4H), 5.99 (bs, 4H), 5.98 (s, 1H), 5.93 (s, 1H), 5.47 (dd, ^3^*J*_H,P_ = 9.1 Hz, ^3^*J*_H,H_ = 5.1 Hz, 1H), 5.27 (ddd, ^3^*J*_H,P_ = 9.4 Hz, ^3^*J*_H,H_ = 4.9 Hz, ^3^*J*_H,H_ = 1.0 Hz, 1H), 5.19 (ddd, ^3^*J*_H,P_ = 9.3 Hz, ^3^*J*_H,H_ = 5.0 Hz, ^3^*J*_H,H_ = 1.6 Hz, 1H), 5.14–5.06 (m, 4H), 4.72 (d, ^3^*J*_H,H_ = 4.9 Hz, 1H), 4.60 (d, ^3^*J*_H,H_ = 5.1 Hz, 1H), 4.58–4.14 (m, 6H), 2.50 (2 x t, ^3^*J*_H,H_ = 7.5 Hz, 4H), 1.64 (h, ^3^*J*_H,H_ = 7.6 Hz, 4H), 1.47–1.14 (m, 16H), 0.89–0.78 (m, 6H); ^13^C{^1^H}-NMR (151 MHz, acetonitrile-d_3_): δ [ppm] = 173.3, 156.9, 153.9, 151.3, 149.4, 140.9, 133.3, 130.6, 130.7, 123.1, 119.8, 93.6, 81.0, 72.7, 71.5, 69.4, 34.7, 32.4, 29.6, 25.5, 23.3, 20.1, 14.3; ^31^P{^1^H}-NMR (162 MHz, acetonitrile-d_3_): δ [ppm] = −4.37, −6.03. MS (ESI-HR): *m*/*z* [M + H]^+^ calcd. for C_25_H_33_N_5_O_8_P^+^: 562.2061, found: 562.2068.

## 4. Conclusions

In sum, although the yields still need improvement, we have identified a flexible and reliable synthesis approach towards membrane-permeable, bio-reversibly masked cNMPs and prepared OB-cNMPs that fulfilled all requirements in terms of synthetic flexibility of the approach, chemical stability, and enzymatic activation to be valuable new tools for the setup of novel cellular assays.
